# A Rapid Prototyping Method for Sub-MHz Single-Element Piezoelectric Transducers by Using 3D-Printed Components

**DOI:** 10.3390/s23010313

**Published:** 2022-12-28

**Authors:** Jinwook Kim, Bryce Menichella, Hanjoo Lee, Paul A. Dayton, Gianmarco F. Pinton

**Affiliations:** Joint Department of Biomedical Engineering, The University of North Carolina at Chapel Hill and North Carolina State University, Chapel Hill, NC 27599, USA

**Keywords:** piezoelectric transducer, ultrasonic transducer, manufacturing, rapid prototyping, additive manufacturing, 3D printing, injection molding

## Abstract

We present a rapid prototyping method for sub-megahertz single-element piezoelectric transducers by using 3D-printed components. In most of the early research phases of applying new sonication ideas, the prototyping quickness is prioritized over the final packaging quality, since the quickness of preliminary demonstration is crucial for promptly determining specific aims and feasible research approaches. We aim to develop a rapid prototyping method for functional ultrasonic transducers to overcome the current long lead time (>a few weeks). Here, we used 3D-printed external housing parts considering a single matching layer and either air backing or epoxy-composite backing (acoustic impedance > 5 MRayl). By molding a single matching layer on the top surface of a piezoceramic in a 3D-printed housing, an entire packaging time was significantly reduced (<26 h) compared to the conventional methods with grinding, stacking, and bonding. We demonstrated this prototyping method for 590-kHz single-element, rectangular-aperture transducers for moderate pressure amplitudes (mechanical index > 1) at focus with temporal pulse controllability (maximum amplitude by <5-cycle burst). We adopted an air-backing design (Type A) for efficient pressure outputs, and bandwidth improvement was tested by a tungsten-composite-backing (Type B) design. The acoustic characterization results showed that the type A prototype provided 3.3 kPa/V_pp_ far-field transmitting sensitivity with 25.3% fractional bandwidth whereas the type B transducer showed 2.1 kPa/V_pp_ transmitting sensitivity with 43.3% fractional bandwidth. As this method provided discernable quickness and cost efficiency, this detailed rapid prototyping guideline can be useful for early-phase sonication projects, such as multi-element therapeutic ultrasound array and micro/nanomedicine testing benchtop device prototyping.

## 1. Introduction

Piezoelectric ultrasound transducers generate acoustic waves by electrical inputs and vice versa. Due to the advantages of fast responses, low cost, and simple structure, piezoelectric transducers have been widely used as useful diagnostic and therapeutic devices in biomedical applications [1,2]. Considerable progress over the past five decades established standardized performance requirements for imaging and therapy transducers: high-frequency (approximately higher than ~5 MHz), low-power (time-averaged intensity, *I*_SPTA_ < 720 mW/cm^2^), and broadband (−6 dB fractional bandwidth, FBW > 50%) for imaging versus low-frequency (<~3 MHz), high-power (peak negative pressure, PNP > 5 MPa), and narrowband (−6 dB FBW < 10%) for therapy [2,3,4,5]. Within these classical categories, standardized piezoelectric ultrasound transducers have been produced by mature fabrication processes and well-packaged transducers are commercially available from third-party manufacturers [6]. However, recent innovations in ultrasound imaging and therapy techniques with theranostic micro/nanomedicines have changed this paradigm [7,8,9,10].

Recent biomedical ultrasound techniques in tumor diagnosis and noninvasive therapies require complex transducer performances out of the classical categorization [11,12,13,14]. Thus, various customized transducers with unique specifications were developed for vascular imaging [15], drug delivery [16], immunotherapy [17], tissue ablation [18], and sonothrombolysis [19]. Although piezoelectric transducer manufacturing is already technically mature and high-end transducer customization is commercially available, long lead time (typically > 8 weeks for manufacturing) with device-packaging costs hindered prompt demonstration of new ultrasound technologies [20]. Conventional prototyping methods typically comprise multiple stages of thickness shaping, stacking, bonding, and housing by using several machining tools that require considerable user experience and long operation time [20,21], which delays the quick demonstration of the new sonication ideas. In the early research phases of applying new sonication ideas, the transducer prototyping quickness is prioritized over the final packaging quality, since the quickness of preliminary demonstration is crucial for promptly determining specific aims, feasible research approaches, and usable sonication parameters, e.g., excitation frequency, peak-negative pressure (PNP), burst duration, and pulse repetition frequency (PRF) [22]. We aim to fill this gap in prototyping methodology by developing a rapid prototyping method for single-element piezoelectric ultrasonic transducers using 3D printing.

3D printing has been a popular manufacturing technique with considerable benefits, such as design flexibility, on-demand production, cost efficiency, rapid prototyping, high spatial resolution (<400 µm), and ease of access [23,24]. Recently, several piezoelectric ultrasound transducers were developed by using 3D printing for biomedical ultrasound applications, such as transcranial drug delivery [25], needle insertion [26], and cavitation therapy [27]. Although these works demonstrated the feasibility of 3D-printable transducer materials as wave generation or propagation media, the flexibility of acoustic properties (i.e., acoustic impedance and attenuation) for optimized matching and backing was still limited compared to conventionally used synthesized epoxy composite materials; acoustic impedances can be tailored by changing volume fractions of high-density (>3000 kg/m^3^) particles.

In this work, we demonstrated a rapid prototyping method by using 3D-printed components that are designed to utilize the injection-molding method for nanoparticle-epoxy composite matching and backing layers with tailored acoustic properties. We provide a detailed explanation of this simplified prototyping method with experimental validation and a discussion of its advantages and limitations compared to conventional transducer fabrication methods.

## 2. Materials and Methods

We demonstrated the manufacturing quality, time, and consistency of our rapid prototyping method via modeling, prototyping, and acoustic testing. We aimed to develop a 590-kHz single-element transducer with an aperture size of 21 mm by 21 mm (8.3λ × 8.3λ, where λ is the wavelength in water). The design objectives were the generation of a megapascal range peak-negative pressure (PNP) amplitude at the far field (approximately further than 21λ from the transducer surface) with moderate bandwidth (−6 dB FBW > 25%) for a short-burst (<5 sinusoidal cycles) excitations aiming for mechanical excitation via controlled cavitation or radiation force with suppressed thermal effects. If a sub-MHz transducer meets these requirements, it enables deep-penetration depth (>50 mm) excitation with temporal controllability within 5-cycle periods for mechanical therapy applications such as transcranial neuromodulation and acoustic angiography [28,29]. As sufficient pressure generation is crucial to induce the desired mechanical effects, we selected an air-backing design (type A) that utilizes the reflected wave amplitudes from the PZT backside yet sacrifices the bandwidth due to a longer ring-down time. To check the feasibility of our rapid prototyping method for wider bandwidth (−6 dB FBW > 40%) applications, we additionally tested the epoxy-composite backing (acoustic impedance > 5 MRayl) design (type B).

### 2.1. Transducer Modeling and Simulation

We used a Krimholtz, Leedom, and Matthaei (KLM) model for 1-D modeling and simulation (BioSono KLM 2.0, Fremont, CA, USA) [30]. 2-D acoustic beam simulation was conducted by using the angular spectrum approach (ASA). The calculation process is described in Appendix A. We considered a flat square aperture source without any focusing effects (zero phases). Feasible materials for transducer components considering experimental validation were used in the simulations. We selected PZT (lead zirconate titanate)-5A as a piezoelectric active material (thickness-mode resonance at 590 kHz), alumina-epoxy composite as a matching layer, and air backing (type A) or tungsten-epoxy as a backing layer (type B) [31]. The key properties of the used materials are listed in Table 1.

### 2.2. Transducer Prototyping

**Figure 1:**
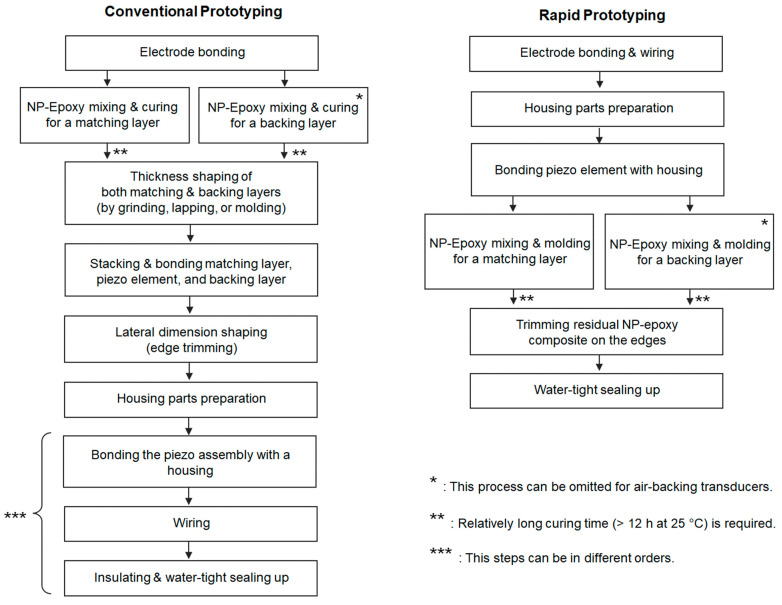
Prototyping flow chart; the rapid prototyping method reduces approximately two core fabrication stages compared to conventional prototyping methods. NP: nanoparticles.

**Figure 2:**
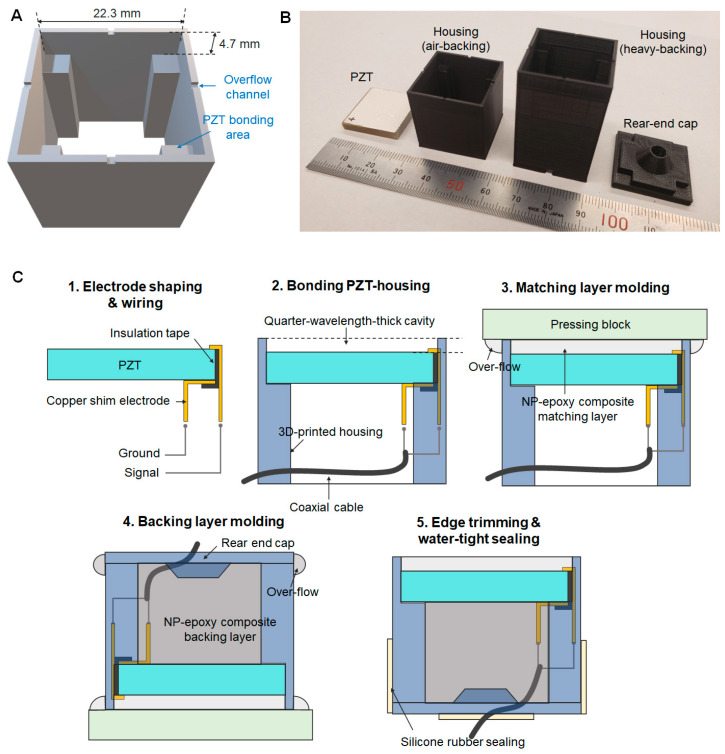
A modeled STL file for 3D-printing (**A**), 3D-printed parts (**B**), and the rapid prototyping procedure (**C**); The stage 4. backing layer molding can be omitted for the air-backing design.

The rapid prototyping procedure comprises three key stages: (1) 3D printing of a transducer outer frame, (2) PZT wiring and bonding to the frame, and (3) matching and backing layers molding directly on the PZT surface. This method is simpler than conventional transducer fabrication methods (Figure 1). The primary factor for this simplification is molding epoxy-composite using a PZT surface and 3D printed part as a mold cavity for a quarter wavelength matching layer, which removes thickness shaping and bonding stages from conventional fabrication methods [32]. We used a 3.5 ± 0.05 mm thick PZT-5A (SM411, STEMiNC, Davenport, FL, USA) plate and alumina-epoxy composite as a matching layer (longitudinal wave speed: 2750 m/s) with a corresponding 590-kHz quarter wavelength thickness of 1.16 mm [31]. Thus, the total thickness of a front cavity in a 3D-printed housing was 4.7 mm (Figure 2A). Since we used the 3D-printed housing as a molding cavity with negligible wave propagation in it, the acoustic properties of 3D-printed material were excluded from design parameters, but its electrical properties (dielectric constant < 5 and dielectric strength > 30 kV/mm) were considered for an electrically insulated housing. We used a fused deposition modeling (FDM)-printer (Ender 3 Pro, Comgrow, Shenzhen, China) with polylactic acid (PLA) filaments as it provides sufficient thickness resolution (100 µm, ~0.02λ of a matching layer material) with high cost-efficiency (8–12 g filaments per each housing assembly).

We started the fabrication procedure by printing the transducer housing and rear-end cap (Figure 2B). The printing parameters are 100-µm deposition height, 800-µm shell thickness, 400-µm top/bottom thickness, 25% fill density, 50-mm/s print speed, 200-°C nozzle temperature, and 50-°C bed temperature. While printing the parts for 90–120 min, we diced the PZT plate into 21 mm × 21 mm square-shaped pieces by using a diamond saw (CH75, Top Tech Machines Co., LTD., Taichung, Taiwan). We bonded copper shim electrodes (25.4 µm-thick, ASTM B152, McMaster Carr, Aurora, OH, USA) on both the top and bottom surfaces of a PZT ceramic using a fast-curing (approximately 1 h) epoxy bond (ClearWeld, J-B Weld, Sulphur Springs, TX, USA). The detailed fabrication process is illustrated in Figure 2C. Once the extended copper electrodes are bonded on the PZT, we soldered a 20 cm 50-ohm coaxial cable (30 AWG, 50CX-41, Temp-Flex, A Molex Co., South Grafton, MA, USA) on the electrodes. The wired PZT ceramic was bonded with the 3D-printed housing using the same fast-curing epoxy bond.

The matching layer molding procedure was initiated by mixing low-viscosity epoxy bond (Epotek301, Epoxy Technology, Inc., Billerica, MA, USA) with alumina powder (25% volume fraction, 0.3-µm particles, Pace Technologies, Tucson, AZ, USA). The mixture was centrifuged (2000 rpm for 3 min) to remove gas bubbles, and approximately 0.6 mL of the mixture was gently transferred to the front cavity surrounded by 3D-printed housing walls and PZT-front-surface cavity floor. We used a pressing block with a flat surface coated by hydrophobic material (e.g., silicone) to produce an undistorted flat surface of the matching layer with easy detachment after the epoxy was fully cured. The epoxy bond is hydrophilic, so the hydrophobicity of a contact surface is crucial to avoid unnecessary bonding of the epoxy composite to a pressing block. To maintain the designed dimensions (i.e., cavity thickness) of the matching layer, we put a 500 g weight on a pressing block during curing (~16 h at room temperature), and we confirmed whether the excessive mixture was squeezed out via overflow channels in each edge.

For the design with a heavy backing (acoustic impedance > 5 MRayl), the rear cavity of the housing was filled with tungsten (11% volume fraction)-epoxy mixture through a similar molding process for the matching layer. The used tungsten powder has an average particle size of 10–20 µm (74MR-0006, Inframat Advanced Materials, Manchester, CT, USA). We skipped this backing layer molding stage for the air-backing design (type A). After a rear-end cap was assembled, external gaps were sealed by room-temperature vulcanizing (RTV) sealant (DOWSIL 732, Dow Corning Corporation, Midland, MI, USA). The cured overflow of matching on the edges was trimmed by using the same diamond saw (CH75). We prototyped three air-backed transducers (A1, A2, and A3) and one heavy-backed transducer (B1) by this rapid prototyping method. We tested the prototyping repeatability for the main transducer design (type A), whereas the type B transducer was tested as a supplementary design to check the availability of wider bandwidth performance.

### 2.3. Transducer Characterization

The prototyped transducers were acoustically characterized by pulse-echo using a high-impedance flat target (stainless steel block with a pressure reflection >94%) and pressure output measurements using a needle hydrophone. The test setups, equipment with manufacturing information, and parameters are summarized in Table 2.

During the pulse-echo testing, we placed a stainless steel block (63 mm × 63 mm × 154 mm) at 98 mm (39λ) away from the transducer aperture, and the transducer was aligned by 3-axis motorized motion stage. Both types of transducers (prototypes A1, A2, A3, and B1) were tested in the same setting. We monitored and acquired echoed signals by the Labview script in a computer with an analog-digital converter. For each measurement, 10 amplitude lines (A-lines) were acquired and averaged to remove the noise signals. We analyzed the normalized pulse-echo sensitivity and −6 dB fractional bandwidth of each prototype transducer using Equation (1), where *f*_*u*_, *f*_*l*_, and *f*_*c*_ denote upper −6-dB frequency, lower −6-dB frequency, and center frequency, respectively. The center frequency *f*_*c*_ is determined by the half of the summation of *f*_*u*_ and *f*_*l*_.

(1) $$-6\;\mathrm{dB}\;\mathrm{FBW}=\frac{f_{u}-f_{l}}{f_{c}}\times 100\;(\%)$$

For pressure output evaluation, we measured the beam profiles of the type A transducer (A1). We assumed that type A and type B transducers have negligible differences in beam profile with the same tone burst input since the aperture shape is identical: a 21 mm (8.3λ) × 21 mm (8.3λ) square shape. The 2D maps on a lateral plane (normal to the wave propagation direction) and an axial plane (parallel to the wave propagation direction) were obtained by hydrophone raster scanning. The beam map size and pixel numbers for mapping in an axial direction were 108 mm (42.8λ) × 30 mm (11.9λ) and 180 × 50 pixels, respectively. For lateral beam mapping, 30 mm (11.9λ) × 30 mm (11.9λ) with 50 × 50 pixels on the plane at a distance of 72 mm (29λ) were scanned. The pixel size was 600 µm × 600 µm which is smaller than a quarter wavelength of 590-kHz waves. We evaluated beam shapes and −3 dB beam width compared to the simulation results.

We measured far-field pressure amplitudes as a function of input burst cycles. This test evaluates whether the transducer can be used as a source element for short-cycle burst (<5 cycles) excitation applications, such as dual-frequency acoustic angiography or histotripsy that requires temporal controllability [18,29,33], since a poorly designed matching layer requires a large number of cycles (>10 cycles) to reach the peak. We positioned the hydrophone tip at 72 mm (29λ) away from the transducer. We changed the cycle numbers of 590-kHz sine bursts from 1 cycle to 10 cycles and acquired corresponding pressure amplitudes while maintaining the voltage amplitude at 134.1 V_pp_. At the same hydrophone position (29λ away), we characterized the transducer pressure amplitude as a function of input voltages that changed from 44.8 V_pp_ to 335.2 V_pp_ with the substeps of 22.4 V_pp_. The number of 590-kHz sine burst cycles was maintained at 10 cycles.

## 3. Results

### 3.1. Modeling and Simulation

**Figure 3:**
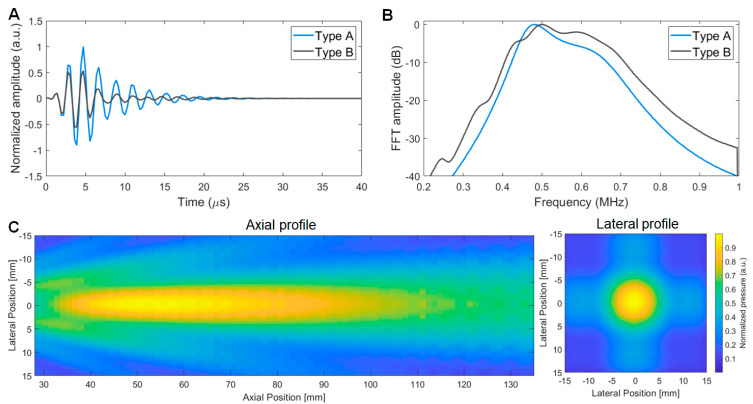
Simulation results: the PE waveform (**A**) and frequency spectra (**B**) of both type A (air−backing) and type B (heavy−backing) transducers, and the beam profile in axial (**left**) and lateral (**right**) planes (**C**).

The 1-D simulation results by using a KLM model exhibited distinctive pulse-echo results between type A and type B transducers (Figure 3). The type A transducer showed a −6 dB fractional bandwidth (FBW) of 31% whereas the type B transducer showed 44%. The normalized pulse-echo (PE) sensitivity of the type A transducer was 1.75-fold of the type B transducer. As we designed the heavy-backing transducer for wider bandwidth performance, the type B transducer showed 1.42-fold −6 dB FBW yet reduced PE amplitude compared to the type A transducer. Figure 3C shows the beam pattern simulation results. In an axial direction, the −3 dB focal length spanned from 12.9λ to 42.7λ. the −3 dB focal width was calculated as 3.5λ at the far-field plane (29λ away from the transducer aperture).

### 3.2. Prototype Transducers

**Figure 4:**
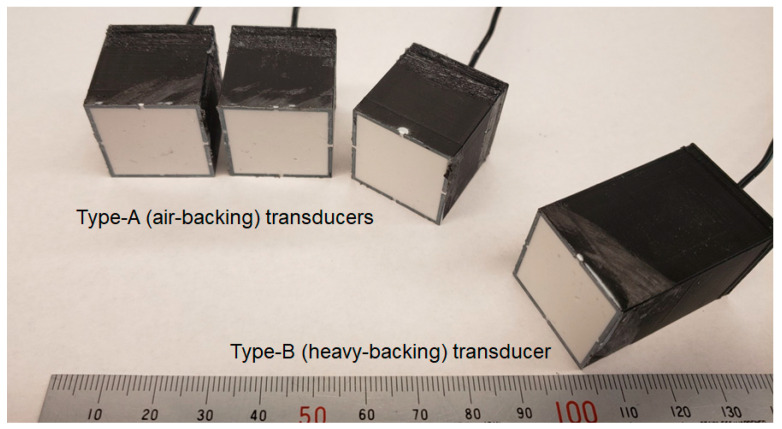
Photo of prototype transducers: type A (air-backing) transducers and a type B (heavy-backing) transducer.

We observed a flat aperture surface of prototyped transducers with an alumina-epoxy composite matching layer (Figure 4) without any visible air bubbles or dents on the transducer surfaces. Electrical resistivity and capacitance were measured by a multimeter to confirm the wire connection, and all prototypes showed expected capacitance (2.6–2.9 nF) considering the PZT size and dielectric constant. This result indicated that the 100 um-thick polytetrafluoroethylene (PTFE) tape provided sufficient electrical insulation.

### 3.3. Pulse Echo Responses

**Figure 5:**
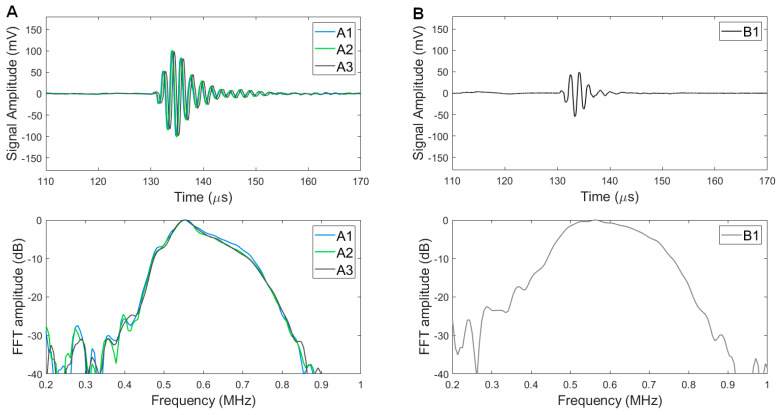
Pulse−echo responses of type A (**A**) and type B (**B**) transducers: waveforms (**upper**) and FFT results (**lower**).

Pulse echo results of the three type A transducers showed −6 dB FBW of 25.3 ± 1.3% and averaged PE amplitude of 226 ± 5 mV (Figure 5A). Although narrower bandwidth than simulation data (31%) was obtained, the PE response showed an acceptable bandwidth considering the performance requirement (−6 dB FBW > 25%). Compared to the type A transducer responses, the type B transducer showed wider bandwidth but lower PE amplitude: 43.3% and 122 mV, respectively (Figure 5B). The type A transducer has a 1.85-fold PE amplitude of the type B transducer, whereas the type B transducer has a 1.71-fold −6 dB FBW of the type A transducer. The discrepancy from the simulation data is possibly caused by (1) mode coupling between the higher harmonic modes of lateral resonance and the fundamental thickness mode (590 kHz) and (2) induced third harmonics (3rd harmonic thickness mode) by the pulsed input since these harmonics were not considered in the KLM models.

We confirmed prototyping consistency using the pulse-echo data of the type A prototype transducers: A1, A2, and A3. As shown in Figure 5A, both the time domain signal and frequency domain signal forms of the three transducers were similar and showed a marginal variation: PE amplitude standard deviation of 5 mV and −6 dB FBW standard deviation of 1.3%. This result indicates that the suggested rapid prototyping method provides fabrication consistency and this method is feasible for multiple-element fabrication with consistent performance for customized sub-MHz array transducers.

### 3.4. Pressure Outputs

**Figure 6:**
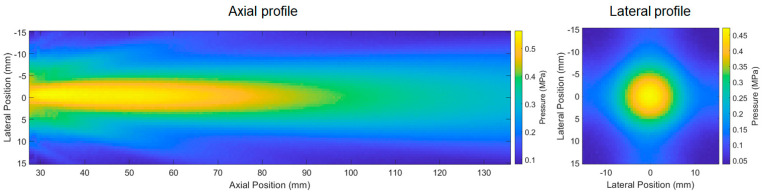
Beam mapping by using the type A transducer, 590 kHz, 10−cycle burst excitation. The lateral profile was mapped at the plane 72 mm (29λ) away from the transducer surface.

The beam profile of the type A transducer (A1) was characterized by hydrophone raster scanning. A −3-dB focal length was observed from 8λ to 38λ (Figure 6). Since the observable amplitude (~10% of the maximum amplitude) of the electrical excitation signal (590-kHz 10-cycle burst) was coupled in the hydrophone signals at the time range from 0 to 17 µs, the near-field −3-dB cut-off end was determined by the additional beam mapping with 5 cycle-burst to avoid the signal distortion by electrically coupled signals. In the lateral direction, the −3 dB focal width was 10.2 mm (4λ). Although the overall shapes of the main lobe, side lobes, and lateral beam profile were similar to the simulation result (Figure 3C), we observed that the measured axial focal zone (8λ to 38λ) was noticeably different from the simulation result (12.9λ to 42.7λ). We considered the possible error factors as excitation bandwidth (single-frequency continuous excitation in ASA simulation vs. 10-cycle excitation with 17% −6-dB FBW in the measurement) and vibration mode differences, i.e., perfectly flat amplitude in ASA vs. mode-coupled vibration effects in a PZT ceramic (21 mm × 21 mm × 3.5 mm) with an aspect ratio (width/thickness) lower than 10. The effects of (1) mode-coupled excitation amplitude profile, (2) effective excitation aperture size, (3) bandwidth effects, (4) dissipation of high-frequency content through a matching layer and water medium, and (5) different mapping grid sizes can be further investigated by 3D finite element analysis.

**Figure 7:**
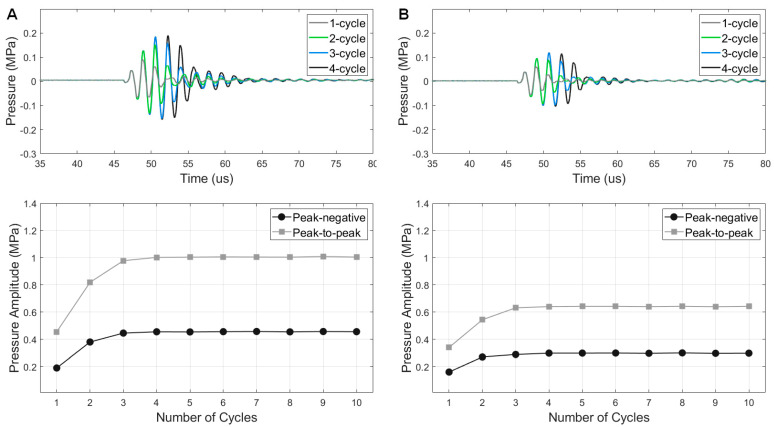
Output-pressure amplitude vs. the number of cycles. Time-domain waveforms of 1−4 burst cycles (**upper**) and the corresponding peak amplitudes (**lower**) of type A transducer (**A**) and type B transducer (**B**).

The measured burst excitation performances exhibited significant differences between the type A transducer and the type B transducer (Figure 7). The PNP amplitude as a function of burst cycle numbers has a similar trend for both type A and type B transducers, but the amplitude values were different due to the considerable wave dissipation by the tungsten-epoxy backing of the type B transducer. By 134.1 V_pp_ excitation, the type A transducer reached a PNP of 455 kPa at 29λ away from the transducer aperture (Figure 7A), whereas the type B transducer showed 299 kPa peak pressure (Figure 7B), which is 66% amplitude of the type A performance. Both types of transducers showed a −12 dB ring-down time of lower than 4.4 µs (<2.6 cycles). Both transducers enable reaching 95% of the maximum pressure by 3-cycle burst excitation and reaching the maximum by 4-cycle excitation. The higher cycle numbers (>5 cycles) maintained the peak pressure amplitudes. This result indicates that the same matching layer design provides similar tone burst wave transmitting forms regardless of the backing layer design, although the absolute pressure amplitude is reduced by the heavy, attenuative backing layer. Thus, the type A transducer design can be suitable for short-burst transmitter elements for ultrasound therapies or multi-frequency harmonic imaging [29]. If receiving performance is required for imaging applications, the type B transducer can be useful due to its wider bandwidth (1.7-fold −6 dB FBW) compared to type A transducers.

**Figure 8:**
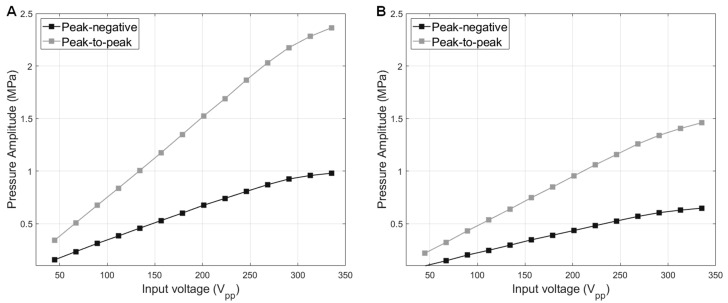
10-cycle burst excitation pressure amplitude vs. input voltages. Both type A transducer (**A**) and type B transducer (**B**) were measured at the same axial distance (29λ away from the transducer surface).

For the measurement point at 29λ away, the transmitting sensitivity of the type A transducer was also discernably higher than the type B transducer: 3.3 kPa/V_pp_ vs. 2.1 kPa/V_pp_ with the voltage input up to 250 V_pp_ (Figure 8). With higher voltage input than 250 V_pp_, the pressure increment was not linear, and the transmitting sensitivity decreased (3.1 kPa/V_pp_ and 2 kPa/V_pp_ for type A and type B transducers, respectively). As we expected for type B transducer design, the attenuative backing layer consumes the backward propagation waves and reduces the output pressure amplitudes. By considering the demonstrated axial pressure data and beam mapping data, we confirmed that the type A transducer generates tone bursts (>5 cycles) with PNP higher than 1 MPa in the axial range from 11.1λ to 27.7λ with 335 V_pp_-input voltage. Considering the depoling voltage limit of PZT-5A (AC 700 V_pp_/mm) and the dielectric breakdown voltage of PTFE film (59 kV/mm), the type A transducer can generate MPa-range pressure by the higher voltage inputs (>335 V_pp_) considering an attenuative acoustic medium and in biomedical applications.

## 4. Discussion

The suggested rapid prototyping method demonstrated the simple, fast, cost-efficient, and consistent manufacturing capability for the customization of sub-MHz, single-element piezoelectric ultrasound transducers. By molding matching and backing layers directly on the PZT surfaces instead of separate shaping and bonding, this rapid prototyping method comprises simpler, reduced fabrication stages than conventional prototyping methods. This simplification provides three advantages in transducer hardware engineering: (1) no complex machining tools required, (2) low material cost, and (3) fast manufacturing. By removing the subtractive thickness shaping process that requires machining tools (e.g., grinder, lapper, bulk molder) with user-experience dependency, the entry barriers for transducer fabrication can be reduced, which potentially facilitates quick demonstration of new sonication ideas associated with micro/nanomedicine excitation.

**Figure 9:**
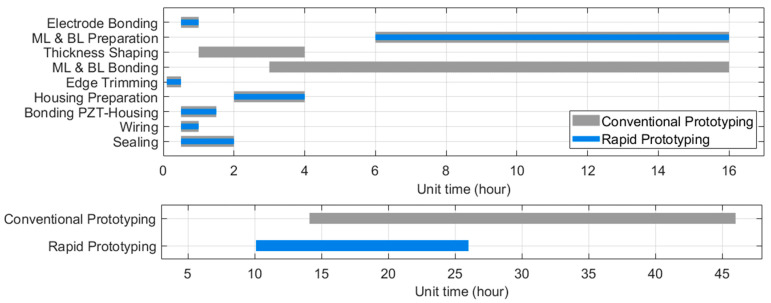
Comparison of estimated manufacturing time of the rapid prototyping method vs. conventional prototyping methods: required time range for each stage (**upper**) and in total (**lower**).

Each fabrication stage in this method requires less than 1 h except for epoxy matching layer curing at room temperature (recommended to wait for 24 h but sufficiently cured within 16 h for handling at the next fabrication stages). A comparison of the exact fabrication time between the reference methods and the rapid prototyping method was not available in this study since a wide variety of fabrication procedures are included in conventional fabrication methods and the completion time of each procedure is affected by equipment setting, required precision level, and user skill levels. For example, our previous works used the same epoxy (Epotek301) and alumina powder (300 nm) as a matching layer that was shaped and bonded by the conventional lapping and stacking procedures. Thickness shaping by a lapper (PM5, Logitech Ltd., Old Kilpatrick, Glasgow, UK) consumed approximately 2 h, and the matching layer bonding required approximately 10 h to complete curing at 60 °C [29,30,34]. We conducted a simplified estimation of the minimum and maximum time required for each procedure based on widely known conventions of transducer manufacturing (Figure 9). This estimation considered the preconditions that the same dimensions and materials (e.g., the same 3D-printed plastic housing) were used for prototyping, which made the ‘ML & BL Bonding’ stage in conventional methods spanned up to 16 h for room-temperature curing of low-viscosity epoxy (e.g., Epotek-301). If the required precision level is low, e.g., thermal deformation during high-temperature (>80 °C) curing is permitted or high-viscosity yet fast-curing (<2 h) epoxy can be used, this process time can be down to 3 h as shown in Figure 9. The used matching and backing molding procedure in the rapid prototyping method consumed approximately 30 min which is significantly shorter than conventional grinding, lapping, or separate molding (0.5–4 h) with the additional bonding process. We estimate that the suggested rapid prototyping method generally reduces more than two fabrication stages (i.e., matching layer shaping, backing layer shaping, and layer stack-and-bonding) from the conventional fabrication methods. Thus, the demonstrated method has six subsequent stages for complete packaging with an approximated required time of 10.1–26 h, whereas the estimated time for typical prototyping methods ranges from 14.1 to 46 h.

The suggested method is simple (reduced two stages from conventional methods), fast (completion time < 26 h), and consistent (PE performance deviation < 6%). However, a couple of noticeable disadvantages of this prototyping method should be clarified. First, since the 3D-printed plastic housing is used from the initial stage to the final stage, any high-temperature (>80 °C) curing process is not suitable for this rapid prototyping method. High temperature (>80 °C) significantly reduces epoxy bond curing time (<6 h at 80 °C), but it also easily deforms the 3D-printed structure made of PLA or photopolymers. If the high-temperature curing polymer (e.g., polydimethylsiloxane, PDMS) is an essential part of the custom transducer design, the quickness of the suggested method can be compromised, e.g., PDMS requires 48 h for curing at 25 °C whereas it is fully cured within 45 min at 100 °C. To overcome this limitation, acrylonitrile butadiene styrene (ABS) can be used for FDM printing as it has good thermal resistance and a high melting point (>100 °C) for fast epoxy curing. However, ABS printing possibly diminishes the sustainability and safety of the rapid prototyping method since ABS is non-biodegradable and toxic when it melts.

Second, here we demonstrated only a single-matching layer design, and the feasibility of adopting multi-matching layer designs for rapid prototyping was not demonstrated. As dual- or triple-matching layer designs are already mature techniques for conventional fabrication methods, the undemonstrated dual-matching feasibility is a limitation of this method. Although multi-layer molding is technically feasible by using a two-stage molding process with additional 3D-printed parts, it possibly compromises fabrication rapidity and simplicity.

Third, we demonstrated only for sub-MHz transducer design which has a large tolerance for fabrication errors compared to general medical ultrasonic frequency ranges (1–10 MHz); typical matching layer materials have a wavelength larger than 2.8 mm for sub MHz range, which can embrace the 100 um manufacturing error as <5% tolerance. For MHz range designs, tens of micron errors in 3D-printed housing (e.g., melted PLA bumps, support structure residue, etc.) possibly cause discernable inconsistent manufacturing quality and performance reduction. We anticipate that the rapid fabrication method for MHz range transducers requires further quality testing between each fabrication stage. In future work, we will evaluate the prototyping capability of this method for MHz-range transducers.

## 5. Conclusions

We demonstrated a rapid prototyping method for 590 kHz single-element piezoelectric transducers. The suggested method simplified the fabrication stages by removing thickness shaping and bonding procedures. By using this method, we produced three air-backed prototype transducers with targeted performances with consistency (performance deviation < 6%). Based on our experimental demonstration in this study, we concluded that the suggested method facilitates rapid prototyping of a reliable sub-MHz square-aperture transducer in less than 26 h. The suggested method will be further investigated to confirm the prototyping feasibility for 1–10 MHz transducer fabrication and batch production of more than 100 identical parts.

## Figures and Tables

**Table 1:** Materials and dimensions of designed transducer parts. V_L_, k_t_, ε/ε_0_, and α denote longitudinal wave speed, thickness-mode coupling coefficient, dielectric constant along the thickness, and attenuation factor.

Structure	Material	Dimensions (mm^3^)	Acoustic Impedance (MRayl)	*V*_L_ (m/s)	*k*_t_	ε/ε_0_	α (dB/cm/MHz)
Drive element	PZT-5A	21 × 21 × 3.5	31.2	4000	0.45	850	-
Matching layer	Alumina (25%)-epoxy	21 × 21 × 1.16	4.8	2750	-	-	5.3
* Backing layer	Tungsten (11%)-epoxy	21 × 21 × 32	5.7	1900	-	-	25

* High-viscosity epoxy (Superclear, FGCI–Fiberglass Coatings, Inc., Petersburg, FL, USA) was used for molding a composite backing layer. For the air-backing design, air density (1.2 kg/m^3^) and wave speed (345 m/s) were used.

**Table 2:** Acoustic characterization setup and parameters.

Test items	Equipment	Parameters
Pulse-echo	Square wave pulser (SP-801A, Ritec, Inc. Warwick, RI, USA) Broadband receiver (BR-640A, Ritec, Inc. Warwick, RI, USA)	Pulser: 0.5 MHz, −12 dB (100 V square wave) Receiver: no gain (0 dB), bandpass 100 kHz–3 MHz, 50-ohm input impedance, digital bandpass filter 100 kHz–3 MHz (5th order Butterworth)
Pressure vs. burst cycles Pressure vs. voltages Pressure beam mapping	Arbitrary function generator RF amplifier (A-150, E&I LTD, Rochester, NY, USA) Hydrophone (HNA-400, Onda Co., Sunnyvale, CA, USA)	Constant parameters: 590 kHz, 20-ms pulse duration Burst cycle test: fixed 134.1-V_pp_ input voltage, varied cycles from 1 to 10 with 1-cycle substep Voltage test: fixed 10 cycles, varied voltages from 44.8 to 335.2 V_pp_ with 22.4-V_pp_ substep Beam mapping: 134.1 V_pp_, 10 cycle

## Data Availability

Not applicable.

## Appendix A

The angular spectrum approach (ASA) provides pressure amplitude and phase information at the propagation planes of interest. The ASA process for beam profile simulations is briefly described here.

As time dependence is assumed as $e^{-j\omega t}$ for harmonic wave propagation, the acoustic pressure wave is expressed as Equation (A1), where $\hat{p}\left(x,y,z\right)$ and $e^{j\Delta \phi \left(x,y,z\right)}$ are harmonic amplitude and phase functions, respectively [35,36].

(A1) $$p\left(x,y,z\right)=\hat{p}\left(x,y,z\right)e^{j\Delta \phi \left(x,y,z\right)}$$

By assuming a uniform propagation medium and assigning *z* as a propagation axis, the angular spectrum of the complex wave at constant *z* is defined by the Fourier transform of p(x,y,z) with respect to *x* and *y* (Equation (A2)), where $k_{x}$ and $k_{y}$ are the wavenumber in the liquid medium along the *x-*axis and *y*-axis, respectively.

(A2) $$P\left(k_{x},k_{y},z\right)=\overset{\infty }{\underset{-\infty }{\int }}\overset{\infty }{\underset{-\infty }{\int }}p\left(x,y,z\right)e^{-j\left(k_{x}x+k_{x}y\right)}dxdy$$

As the amplitude and phase maps of the transducer aperture plane (*z* = 0) are known, $P\left(k_{x},k_{y},0\right)$ can be defined, e.g., a 21 mm (8.3λ) × 21 mm (8.3λ) square uniform amplitude with a zero phase (flat surface) map in this study. By using known values at *z* = 0 in the Fourier domain, $P\left(k_{x},k_{y},0\right)$, angular spectrum at any propagation planes can be calculated by multiplying a propagation function, $H\left(k_{x},k_{y},z\right)$ as shown in Equation (A3), where the propagation function can be written as Equation (A4).

(A3) $$P\left(k_{x},k_{y},z\right)=P\left(k_{x},k_{y},0\right)H\left(k_{x},k_{y},z\right)$$

(A4) $$H\left(k_{x},k_{y},z\right)=e^{jz\sqrt{k^{2}-k_{x}^{2}-k_{y}^{2}}}$$

Once the angular spectrum at a plane of interest is calculated, the real space pressure field at the same plane can be calculated by the inverse Fourier transform as shown in Equation (A5).

(A5) $$p\left(x,y,z\right)=\frac{1}{4\pi^{2}}\overset{\infty }{\underset{-\infty }{\int }}\overset{\infty }{\underset{-\infty }{\int }}P\left(k_{x},k_{y},z\right)dk_{x}dk_{y}$$

We followed this process to simulate 590-kHz wave propagation from a 21 mm (8.3λ) × 21 mm (8.3λ) square-aperture transducer. To avoid higher spatial frequency reflection effects in the calculations, we assigned a sufficiently large (190 mm × 190 mm in lateral dimensions) axial plane (normal to the propagation direction, *z*-axis) for the ASA calculation. The pixel size was 316 µm (190 mm/601 pixels) which was λ/8 for 590-kHz waves.
